# How many species of *Apodemus* and *Rattus* occur in China? A survey based on mitochondrial cyt *b* and morphological analyses

**DOI:** 10.24272/j.issn.2095-8137.2018.053

**Published:** 2018-06-19

**Authors:** Shao-Ying Liu, Kai He, Shun-De Chen, Wei Jin, Robert W. Murphy, Ming-Kun Tang, Rui Liao, Feng-Jun Li

**Affiliations:** 1 Sichuan Academy of Forestry, Chengdu Sichuan 610081, China; 2 Kunming Institute of Zoology, Chinese Academy of Sciences, Kunming Yunnan 650223, China; 3 College of Life Sciences, Sichuan Normal University, Chengdu Sichuan 610066, China; 4 Centre for Biodiversity and Conservation Biology, Royal Ontario Museum, Toronto M5S 2C6, Canada

**Keywords:** Alpha diversity, *Apodemus*, DNA-barcoding, *Rattus*, Taxonomy, Phylogenies, New subspecies

## Abstract

*Apodemus* (mice) and *Rattus* (rats) are the top rodent reservoirs for zoonoses in China, yet little is known about their diversity. We reexamined the alpha diversity of these two genera based on a new collection of specimens from China and their cyt *b* sequences in GenBank. We also tested whether species could be identified using external and craniodental measurements exclusively. Measurements from 147 specimens of *Apodemus* and 233 specimens of *Rattus* were used for morphological comparisons. We analysed 74 cyt *b* sequences of *Apodemus* and 100 cyt *b* sequences of *Rattus* to facilitate phylogenetic estimations. Results demonstrated that nine species of *Apodemus* and seven species of *Rattus*, plus a new subspecies of *Rattus nitidus*, are distributed in China. Principal component analysis using external and craniodental measurements revealed that measurements alone could not separate the recognized species. The occurrence of *Rattus pyctoris* in China remains uncertain.

## INTRODUCTION

Small volant and nonvolant mammals are important components of ecological communities and play vital roles in ecological systems. They are among the most common agents for infections and, thus, have strongly affected human history. For example, black rats (*Rattus rattus*) are considered likely agents for the spread of Oriental rat fleas, which drove the Black Death plague throughout Europe and the Mediterranean during the 14th century and killed 30%–60% of the European population (Barnett, 2001; Duplantier et al., 2003). More recent examples of small mammal zoonoses include severe acute respiratory syndrome (SARS) caused by a coronavirus and Ebola hemorrhagic fever caused by *Ebolavirus*, with hosts including, but not limited to, bats and civets (Klein & Calisher, 2007; Menachery et al., 2015). Rodent-borne diseases such as plague and hantavirus have made considerable contributions to human illnesses and are responsible for more deaths than all wars combined (Klein & Calisher, 2007). New pathogens, especially hantaviruses, have been isolated from rodents in China and adjacent countries annually (Huang et al., 2017). Because different species have specific immune systems and different levels of tolerance to zoonotic infections, identification of rodent reservoirs of zoonotic pathogens is a high priority (Meerburg et al., 2009).

Rats and mice often top the zoonoses reservoir list of the Chinese Center for Disease Control and Prevention (China CDC) because of the large number of species, substantial population sizes, and high potential for carrying zoonotic pathogens (Wu et al., 2017). Unfortunately, we still do not know how many species of rats and mice occur in China, or which species carry what pathogens, even for the most common genera such as *Apodemus* and *Rattus*. The reasons for this are complicated. Both *Apodemus* and *Rattus* have complex evolutionary and taxonomic histories, with classifications continuously being updated. Switching between valid species and synonyms causes considerable confusion, especially for non-specialist researchers. Furthermore, many species occur only in remote mountains or near national borders with high species diversity, such as Yunnan, Xizang (Tibet), and Xinjiang. Indeed, the rats and mice of southern Xizang and western Xinjiang remain to be studied carefully. Finally, many rodents are difficult to identify to species level due to the number of morphologically similar species (Galan et al., 2012).

The latest version of Mammal Species of the World (Musser & Carleton, 2005) recognized 20 species of *Apodemus* and 162 synonyms. Several scenarios for the classification of *Apodemus* have been proposed (Filippucci, 1992; Martin et al., 2000; Musser et al., 1995; Serizawa et al., 2000; Zimmermann, 1962), but none are strongly supported, and phylogenies remain poorly resolved despite molecular efforts (Liu et al., 2004; Serizawa et al., 2000; Suzuki et al., 2003). Furthermore, the number of species in China remains unknown, with previous estimations varying from six (Corbet, 1978; Xia, 1984), seven (Liu et al., 2002; Liu et al., 2004), eight (Smith et al., 2008), and nine species (Nowak, 1999; Wang, 2003). Many authors have suggested that *A. sylvaticus* occurs in Xinjiang, China (Corbet, 1978; Xia, 1984; Wang, 2003), whereas others have argued that the species is *A. uralensis* (Smith et al., 2008). The former species occurs in Western Europe (Bousbouras, 1999; Macholán et al., 2001; Mezhzherin & Zykov, 1991; Michaux et al., 1996), and its incorrect identification in China likely relates to outdated taxonomy.

*Rattus*, another problematic genus, has had 25 subgenera and more than 550 species and subspecies named (Simpson, 1945). Currently, 66 species are recognized but uncertainty persists. Previous supermatrix analysis did not obtain a monophyletic *Rattus*, indicating that systematics is far from resolved (Steppan & Schenk, 2017). Arguments also persist for the most common species, including black rats whose species boundary remains unfixed (Aplin et al., 2011). The number of species of *Rattus* in China is also uncertain and varies from four (Corbet, 1978), seven (Smith et al., 2008), and nine (Wang, 2003).

Similar to other rodents, species in these two genera are difficult to identify or distinguish morphologically due to their similar appearance, overlapping measurements, and key factors involving the single cusp on their teeth. Diagnosis often requires clean skulls, which are not always available or correctly prepared. DNA barcoding is a promising approach but requires a solid reference database (Moritz & Cicero, 2004). Unfortunately, GenBank data are problematic because many rodent sequences are uploaded by non-specialists such as epidemiological researchers. This reduces the reliability of environmental assessment reports and hampers our understanding of host and disease associations.

Herein, we revisited the alpha diversity of *Apodemus* and *Rattus* in China based on a collection of more than 400 specimens and the integration of cyt *b* sequences. We evaluated the species of both genera in China and assessed if they could be identified easily using traditional morphometric approaches.

## MATERIALS AND METHODS

### Morphological diagnoses and analyses

We examined 147 specimens of *Apodemus* and 233 specimens of *Rattus* collected from multiple localities across China. External and skull measurements followed Liu et al. (2012). External measurements of fresh specimens in the field were taken to the nearest 0.5 mm using a steel tape. These included head-body length (HBL), hind-foot length (HFL), ear length (EL) and tail length (TL) (museum specimens from original records). We measured eight skull variables using a digital caliper graduated to the nearest 0.01 mm from 147 intact skulls of *Apodemus* and 233 intact skulls of *Rattus*, including greatest length of skull (SGL), nasal bone length (NBL), zygomatic breadth (ZB), skull basal length (SBL), upper toothrow length (UTRL), upper molar row length (UMRL), auditory bulla length (ABL), and mandible length (ML). Examined specimens (Supplementary Table S1) were deposited in the Kunming Institute of Zoology (KIZ), Sichuan Academy of Forestry (SAF), Beijing Institute of Zoology (BIZ), Guangdong Key Laboratory of Animal Conservation and Resource Utilization, and Fujian Center for Disease Control Prevention.

Specimens were roughly identified based on external and craniodental morphology, following Kaneko (2010) and Smith et al. (2008). External and craniodental measurements largely overlapped between species (see Results) and were inadequate for identification. However, several diagnostic characters on the upper molars were constructive in classification, including the number of lingual angles of the first and second upper molar, presence/absence of cusp t3 on the first upper molar, and numbers of internal lobes on the third upper molars. We also cross-checked results based on morphological diagnoses with molecular sequences (when available) to refine identification. All specimens were identified by the same researcher (SYL) for consistency. We finally assigned our specimens to nine species of *Apodemus*, seven species of *Rattus*, and a new subspecies of *Rattus nitidus*, respectively.

We analyzed morphometric variation using principal component analyses (PCAs) on log10-transformed variables using two datasets for each species. The first dataset included both external and craniomandibular variables, whereas the second dataset included craniomandibular variables only. Inclusion of the external data tested whether these measurements could increase the accuracy of identification. Statistical analysis was performed using SPSS v16.0 (SPSS Inc., USA). When two or more recognized species were not well separated in the principal component (PC) plots, analysis of variance (ANOVA) was applied to analyze among group differences.

### Molecular analysis

**Figure 1:**
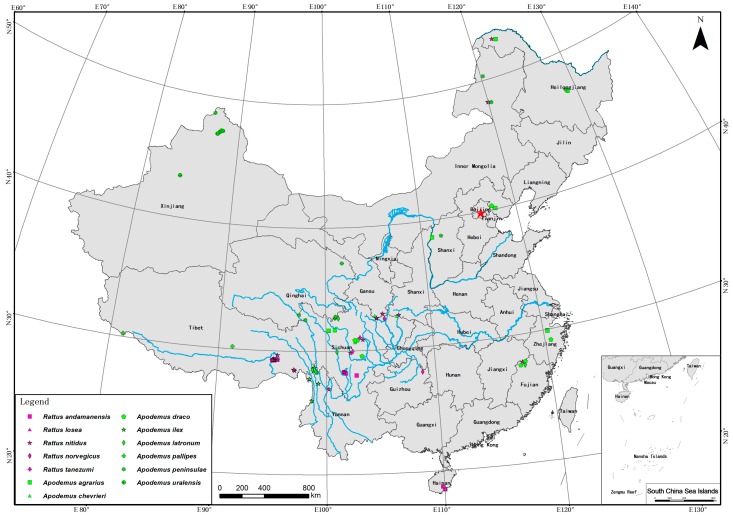
Localities of molecular samples from China in this study

We sequenced mitochondrial cyt *b* for 74 and 100 specimens of *Apodemus* and *Rattus*, respectively. Localities of molecular samples used from China are mapped in Figure 1. All sequenced specimens were deposited in the SAF. Total genomic DNA was extracted using the standard phenol-chloroform method (Sambrook & Russell, 2001). We used the universal primers of mammalian cyt *b* L14724/H15915 for amplification (Irwin et al., 1991). Polymerase chain reaction (PCR) was conducted in a 25-µL reaction volume, including 2.5 µL of 10×EX Taq buffer (Mg^2+^ Free), 2 µL of 2.5 mmol/L dNTP, 1.5 µL of 25 mmol/L MgCl_2_, 1 µL of 10 µmol/L primers, and 1 unit of EX Taq polymerase (TaKaRa Biotech, Dalian, China). The product was purified using an EZNA^TM^ Gel Extraction Kit (Omega, USA), and was sequenced using the same primers for amplification on an ABI 3730XL sequencer. Sequences were assembled and edited using SeqMan and EditSeq (DNASTAR, Lasergene v7.1) before subsequent analyses.

To avoid misidentification, we first conducted a “naïve identification” for the obtained sequences using the “identify organism” workflow in Geneious v11 (Biomatters, New Zealand). The software blasted each sequence against the GenBank nucleotide collection (nr/nt) database. When pairwise identity between the query (our sequence) and subject (in GenBank) sequences was higher 98%, Geneious considered them as the same species. We cross-checked the results of both morphological and molecular identifications, and when the identification was inconsistent, we revisited the skin and skull specimens before applying an identity.

To provide a better picture of species diversity in China, we downloaded cyt *b* sequences of *Apodemus* (*n*=477) and *Rattus* (*n*=273) in China from GenBank, discarding sequences shorter than 800 bp. We also included cyt *b* data representing another 12 species of *Apodemus* and 14 species of *Rattus* from outside of China. An additional five sequences of *R. pyctoris* from Nepal were included. For better estimation of phylogenetic relationships, we downloaded the mitochondrial genomes (mitogenomes) of seven species of *Apodemus* and 14 species of *Rattus* (Supplementary Table S2). One mitogenome under the name of “*Apodemus chejuensis*” may not have been a valid species. Cyt *b* of *Tokudaia* spp. (*n*=3) and a mitogenome of *Bandicota indica* were selected as outgroup representatives for *Apodemus* and *Rattus*, respectively, following Steppan & Schenk (2017). In total, the datasets for *Apodemus* and *Rattus* included 572 (with seven mitogenomes) and 397 sequences (15 mitogenomes), respectively. We aligned the sequences for each genus using MAFFT v7.3 implemented in Geneious v11. We removed all tRNAs, D-loop, and *ND6* sequences from the alignments, and only used rRNAs and 13 protein-coding genes for phylogenetic analyses. Sequence genetic distances were calculated for cyt *b* using MEGA v.5 (Tamura et al., 2011) under the Kimura 2-parameter model (Kimura, 1980).

### Phylogenetic analyses

We employed RAxML v8.2.10, a maximum likelihood-based approach, for phylogenetic analyses. We partitioned the alignments by genes, except for cyt *b*, which we partitioned into the 1^st^+2^nd^ and 3^rd^ codon positions. Analyses were performed on the CIPRES Science Gateway. We used GTR+G as the evolutionary model for each partition because RAxML does not accept models other than GTR or GTR+G. We ran each analysis using the rapid bootstrapping algorithm and let RAxML halt bootstrapping automatically. We also repeated analyses using alternative strategies, such as different partitioning schemes (e.g., partitioned by gene and codon positions for all coding genes) and evolutionary models (e.g., using GTR model instead of GTR+G), none of which strongly altered phylogenetic relationships (i.e., different relationships supported by bootstrap values (BS) ≥75)).

## RESULTS

### Morphological analysis

#### Morphological analysis of *Apodemus*

**Figure 2:**
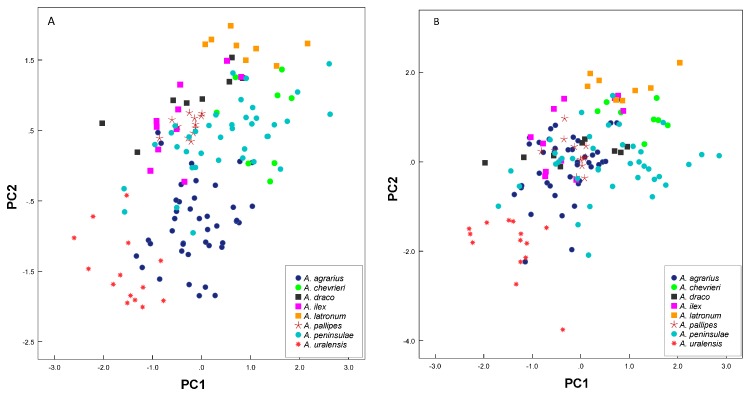
Principal component analysis of the first three principal components among the eight species of *Apodemus* based on both external and craniomandibular variables (A) and craniomandibular variables only (B)

Morphological measurement statistics of the eight *Apodemus* species, excluding *A. semotus*, are given in Table 1. In the first PCA, using all 12 measurements (*n*=139), the first and second principal components accounted for 57.6% (eigenvalue=6.9; Table 2, a) and 11.7% (eigenvalue=1.4) of total variation, respectively, with all other principal components having eigenvalues smaller than 1. PC1 was positively correlated with all craniodental variables (loadings>0.63), and PC2 was positively correlated with external measurements (loadings>0.55). The PC1 and PC2 plot (Figure 2A) did not clearly separate the species. *Apodemus latronum* plotted on the positive regions of PC1 and PC2, indicating a large body, long tail, long hindfeet, and long ears. In accordance with its small skull and small external measurements, *A. uralensis* occurred along the negative regions of PC1 and PC2. The sister- or closely related species *A. agrarius* and *A. chevrieri* as well as *A. pallipes* and *A. uralensis* were well separated, but both pairs overlapped with *A. peninsulae*, *A. draco*, and *A. ilex*, which, in turn, largely overlapped. For the second PCA, using eight craniodental measurements (*n*=141), the first principal component accounted for 69.5% of variation (eigenvalue=5.6; Table 2, b). The other principal components accounted for less than 9.4% (eigenvalue≤0.75) of total variation, indicating they were not stable (Shankardass, 2000). Seven variables were positively correlated with PC1 (loading>0.56), except for UMRL (loading=0.076), which was positively correlated with PC2 (loading=0.93). The PC1 and PC2 plot (Figure 2B) was similar to the previous plot. None of these species were clearly separated from all others. *Apodemus chevrieri* and *A. latronum* plotted on the positive regions of PC1, indicating a relatively large skull, and *A. uralensis* occurred along the negative region of PC1 in accordance with its small skull.

**Table 1:** Measurement statistics of *Apodemus*

		**SGL**				**NBL**				**ZB**				**SBL**			
	***n***	**Mean**	***SD***	**Minimum**	**Maximum**	**Mean**	***SD***	**Minimum**	**Maximum**	**Mean**	***SD***	**Minimum**	**Maximum**	**Mean**	***SD***	**Minimum**	**Maximum**
*A. latronum*	15	28.03	2.54	20.23	30.86	11.12	0.60	9.70	12.17	13.41	0.67	12.38	14.96	25.21	1.58	22.37	27.52
*A. peninsulae*	38	27.76	1.60	24.31	31.75	10.64	1.02	8.09	12.42	13.49	0.96	12.01	15.35	25.58	1.87	22.50	29.81
*A. chevrieri*	8	29.02	0.83	27.83	29.99	10.65	0.74	9.16	11.63	13.63	0.34	13.06	14.02	26.29	1.24	24.47	27.57
*A. agrarius*	41	26.57	0.97	24.55	28.51	9.55	0.53	8.50	10.67	12.56	0.52	11.23	13.34	24.65	1.20	20.82	26.73
*A. ilex*	10	26.63	1.25	25.31	28.88	10.10	0.79	9.13	11.44	12.61	0.37	12.09	13.31	23.42	1.19	22.17	25.45
*A. draco*	9	26.80	1.63	23.83	29.19	10.56	1.06	8.65	11.80	12.59	0.52	11.83	13.27	23.64	1.59	20.97	25.41
*A. pallipes*	12	26.84	0.58	25.76	27.98	10.37	0.37	9.45	10.95	12.73	0.26	12.26	13.13	23.63	0.56	22.37	24.30
*A. uralensis*	14	24.37	0.89	22.49	25.45	8.72	0.48	7.96	9.74	12.38	0.52	11.68	13.20	22.66	0.96	20.49	23.82
		**UTRL**				**UOSL**				**TBL**				**ML**			
	***n***	**Mean**	***SD***	**Minimum**	**Maximum**	**Mean**	***SD***	**Minimum**	**Maximum**	**Mean**	***SD***	**Minimum**	**Maximum**	**Mean**	***SD***	**Minimum**	**Maximum**
*A. latronum*	15	14.41	0.76	12.94	15.60	5.00	0.23	4.47	5.39	5.52	0.35	4.90	6.38	10.48	3.58	6.16	14.41
*A. peninsulae*	38	13.71	0.94	11.52	15.82	4.27	0.29	3.41	5.10	5.34	0.40	4.53	6.17	13.25	1.11	11.02	15.79
*A. chevrieri*	8	14.55	0.64	13.57	15.52	4.59	0.23	4.24	4.91	5.90	0.25	5.52	6.19	14.61	0.67	13.70	15.74
*A. agrarius*	41	12.97	0.73	10.99	14.64	4.18	0.27	3.45	4.84	5.22	0.31	4.55	5.69	12.61	0.70	10.28	14.06
*A. ilex*	10	12.74	0.66	11.98	13.95	4.42	0.37	3.97	4.97	5.33	0.25	4.90	5.73	13.21	0.83	11.97	14.97
*A. draco*	9	13.10	0.72	11.98	14.03	4.24	0.06	4.15	4.35	5.23	0.35	4.50	5.62	13.03	1.09	11.36	14.32
*A. pallipes*	12	13.25	0.32	12.73	13.83	4.34	0.16	4.14	4.67	4.83	0.13	4.62	5.12	13.51	0.40	12.98	14.10
*A. uralensis*	14	11.23	0.47	10.51	12.00	3.60	0.25	2.89	3.82	4.68	0.29	4.30	5.23	11.57	0.50	10.50	12.28
		**HBL**				**TL**				**HFL**				**EL**			
	***n***	**Mean**	***SD***	**Minimum**	**Maximum**	**Mean**	***SD***	**Minimum**	**Maximum**	**Mean**	***SD***	**Minimum**	**Maximum**	**Mean**	***SD***	**Minimum**	**Maximum**
*A. latronum*	15	100.90	9.84	88.00	115.00	104.27	8.41	90.00	122.00	24.17	0.98	22.50	26.00	20.20	0.80	19.00	22.00
*A. peninsulae*	38	100.32	11.96	73.00	137.00	94.26	10.96	72.00	121.00	22.95	1.59	18.00	25.00	16.08	1.58	14.00	21.00
*A. chevrieri*	8	102.50	8.78	90.00	113.00	94.12	9.40	82.00	105.00	23.63	1.69	21.00	26.00	16.12	1.89	13.00	18.00
*A. agrarius*	41	100.17	8.28	85.00	120.00	82.54	11.55	50.00	101.00	19.55	1.86	14.00	24.00	13.39	1.05	12.00	16.00
*A. ilex*	10	86.30	5.83	80.00	98.00	92.80	11.24	72.00	107.00	21.90	1.10	20.00	24.00	17.50	1.43	15.00	19.00
*A. draco*	9	92.11	9.85	75.00	103.00	105.11	12.08	84.00	119.00	48.89	77.30	21.00	255.00	17.33	0.71	16.00	18.00
*A. pallipes*	12	93.75	3.93	87.00	99.00	95.67	4.14	90.00	104.00	21.58	0.67	21.00	23.00	16.58	0.52	16.00	17.00
*A. uralensis*	14	83.86	7.31	67.00	94.00	74.36	4.99	65.00	83.00	19.82	1.14	18.00	22.00	12.11	2.65	9.00	17.00

Abbreviations are explained in the Materials and Methods section. All measurements are in mm.

**Table 2:** Factor loadings and percentage of variance explained for principal component analysis

	*Apodemus*				*Rattus*			
	a		b		c		d	
Variables	PC1	PC2	PC1	PC2	PC1	PC2	PC1	PC2
SGL	0.82	0.07	0.83	0.08	0.96	–0.05	0.97	–0.03
NBL	0.64	0.39	0.84	0.08	0.89	–0.06	0.90	–0.03
ZB	0.83	0.00	0.99	–0.24	0.92	–0.08	0.92	–0.08
SBL	1.01	–0.17	0.95	–0.09	0.97	–0.08	0.97	0.01
UTRL	0.80	0.23	0.72	0.37	0.95	–0.16	0.97	–0.19
UMRL	0.29	0.55	0.08	0.93	0.71	0.15	0.71	–0.35
ABL	0.77	0.00	0.56	0.34	0.68	0.14	0.70	0.65
ML	0.64	0.24	0.71	0.17	0.92	–0.14	0.93	0.02
HBL	0.69	–0.09	N/A	N/A	0.79	–0.25	N/A	N/A
TL	0.13	0.74	N/A	N/A	0.67	0.52	N/A	N/A
HZL	–0.04	0.82	N/A	N/A	0.74	–0.24	N/A	N/A
EL	–0.09	0.94	N/A	N/A	0.40	0.84	N/A	N/A
Eigenvalues	6.9	1.4	5.60	0.75	7.99	1.21	6.32	0.57
Total variance explained (%)	57.6	11.7	69.50	9.40	66.54	10.08	79.05	7.08

For abbreviations see Materials and Methods. N/A: Not available.

One-way analysis of variance (ANOVA) revealed that the seven species differed significantly (*P*<0.05) in all external and cranial characters tested, except for NBL (*P*=0.497), TL (*P*=0.064), and HFL (*P*=0.094). Results showed significant differences as follows: UTRL, MRL, ABL, and ML between *A. peninsulae* and *A. chevrieri*; ZB, SBL, UTRL, HBL, and EL between *A. peninsulae* and *A. ilex*; SGL, ZB, SBL, HBL, TL, HFL, and EL between *A. peninsulae* and *A. draco*; ZB, SBL, and ABL between *A. peninsulae* and *A. pallipes*; SGL, ZB, SBL, UTRL, ABL, ML, HBL, and EL between *A. chevrieri* and *A. ilex*; SGL, ZB, SBL, MRL, UTRL, ABL, ML, HBL, TL, and HFL between *A. chevrieri* and *A. draco*; SGL, ZB, SBL, UTRL, MRL, ABL, and ML between *A. chevrieri* and *A. pallipes*; TL and HFL between *A. ilex* and *A. draco*; and ABL, TL, and HFL between *A. draco* and *A. pallipes*. Thus, morphological analysis indicated that the eight species of *Apodemus* could be separated by the 12 morphological characters, validating the taxonomic status of these species in China.

#### Morphological analysis of *Rattus*

**Figure 3:**
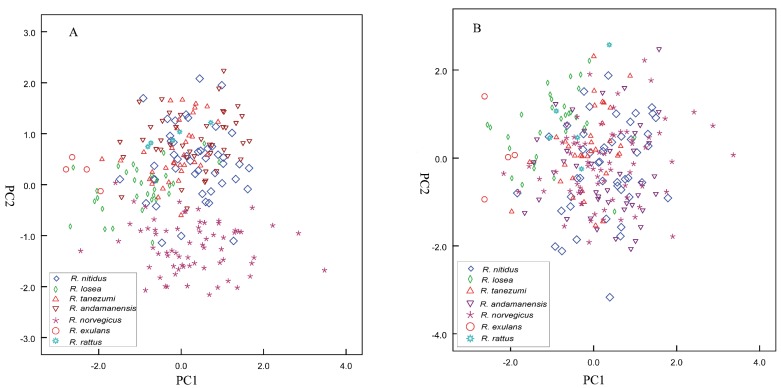
Principal component analysis of the first three principal components among the seven species of *Rattus* based on both external and craniomandibular variables (A) and craniomandibular variables only (B)

Morphological measurement statistics of the seven species of *Rattus* and a putatively new subspecies of *R. nitidus* (from southern Xizang) are given in Table 3. In the first PCA, which used all 12 measurements (*n*=233), the first and second principal components accounted for 66.54% (eigenvalue=7.99) and 10.08% (eigenvalue=1.21) of total variation, respectively (Table 2, c), with all other principal components having eigenvalues smaller than 1. Most species largely overlapped (Figure 3A). In the second PCA, which used eight craniodental measurements (*n*=233), the first and second principal components accounted for 79.05% (eigenvalue=6.32) and 7.08% (eigenvalue=0.57) of total variation, respectively (Table 2, d). The PC1 and PC2 plot (Figure 3B) revealed largely overlapping species.

**Table 3:** Measurement statistics of *Rattus*

		**SGL**				**NBL**				**ZB**				**SBL**			
	***n***	**Mean**	***SD***	**Minimum**	**Maximum**	**Mean**	***SD***	**Minimum**	**Maximum**	**Mean**	***SD***	**Minimum**	**Maximum**	**Mean**	***SD***	**Minimum**	**Maximum**
*R. nitidus*	46	41.93	3.22	28.40	47.05	16.13	1.68	10.54	19.00	19.87	1.16	17.64	22.30	38.30	2.36	32.82	43.00
*R. nitidus nitidus*	31	41.62	3.53	28.40	47.05	15.90	1.75	10.54	18.66	19.66	1.11	17.64	21.75	37.96	2.30	32.82	41.82
*R. nitidus thibetanus*	15	42.56	2.44	38.39	45.85	16.62	1.47	14.02	19.00	20.31	1.15	18.63	22.30	38.99	2.40	35.19	43.00
*R. losea*	31	37.12	3.32	31.24	46.53	13.51	1.77	10.06	18.35	17.99	1.38	15.02	21.12	34.37	2.61	29.72	40.55
*R. tanezumi*	34	40.11	2.06	33.98	43.27	14.50	1.26	11.69	16.57	19.57	1.03	16.73	21.67	36.50	1.96	30.75	39.80
*R. andamanensis*	44	41.49	3.06	34.87	46.80	15.13	1.75	11.25	17.84	20.33	1.63	16.88	24.05	37.69	2.93	31.76	42.72
*R. norvegicus*	69	41.63	3.52	31.75	52.13	15.33	1.69	10.79	20.22	20.42	1.88	15.33	25.63	38.16	3.30	29.95	48.56
*R. exulans*	4	32.86	2.15	31.09	35.88	11.33	1.64	9.91	13.14	15.77	0.73	15.15	16.79	30.49	1.22	29.15	32.04
*R. rattus*	5	39.86	2.10	37.96	43.15	14.11	1.24	12.93	16.02	18.83	0.71	17.93	19.58	37.75	2.18	35.75	41.03
		**UTRL**				**UMRL**				**ABL**				**ML**			
	***n***	**Mean**	***SD***	**Minimum**	**Maximum**	**Mean**	***SD***	**Minimum**	**Maximum**	**Mean**	***SD***	**Minimum**	**Maximum**	**Mean**	***SD***	**Minimum**	**Maximum**
*R. nitidus*	46	21.29	1.38	18.62	24.36	7.39	0.54	6.15	8.92	7.36	0.56	6.03	8.32	21.70	2.02	16.41	26.17
*R. nitidus nitidus*	31	20.96	1.25	18.62	23.02	7.29	0.53	6.15	8.35	7.28	0.59	6.03	8.32	21.08	1.85	16.41	24.65
*R. nitidus thibetanus*	15	21.97	1.45	19.56	24.36	7.62	0.51	6.66	8.92	7.54	0.42	6.86	8.11	22.96	1.78	19.81	26.17
*R. losea*	31	18.95	1.62	15.86	21.97	6.53	0.42	5.79	7.63	7.17	0.40	6.54	7.84	19.85	1.66	15.76	22.69
*R. tanezumi*	34	20.13	1.22	16.76	22.47	7.38	0.38	6.39	8.18	7.45	0.51	6.22	8.62	20.76	1.09	17.81	22.59
*R. andamanensis*	44	21.36	1.73	17.89	24.02	7.55	0.62	6.11	8.70	7.39	0.52	6.25	8.89	21.70	1.84	17.70	24.65
*R. norvegicus*	69	21.66	1.93	17.01	27.14	7.35	0.47	6.32	8.55	7.40	0.56	6.51	8.68	22.02	2.33	17.02	29.63
*R. exulans*	4	16.87	0.87	15.73	17.84	6.12	0.45	5.56	6.51	6.51	0.26	6.15	6.73	16.24	0.94	15.21	17.49
*R. rattus*	5	19.61	0.83	18.83	20.88	6.73	0.38	6.41	7.39	7.41	0.48	6.99	8.24	19.48	0.98	18.43	20.70
		**HBL**				**TL**				**HFL**				**EL**			
	***n***	**Mean**	***SD***	**Minimum**	**Maximum**	**Mean**	***SD***	**Minimum**	**Maximum**	**Mean**	***SD***	**Minimum**	**Maximum**	**Mean**	***SD***	**Minimum**	**Maximum**
*R. nitidus*	46	164.59	18.34	123.00	205.00	168.09	18.38	131.00	210.00	34.14	2.12	30.00	40.00	23.25	2.21	18.00	28.00
*R. nitidus nitidus*	31	161.32	19.18	123.00	205.00	161.81	16.71	131.00	192.00	33.79	1.89	30.00	37.00	22.96	2.16	18.00	27.00
*R. nitidus thibetanus*	15	171.33	14.84	155.00	205.00	181.07	14.81	162.00	210.00	34.87	2.45	31.00	40.00	23.87	2.26	19.00	28.00
*R. losea*	31	152.07	20.82	103.00	192.00	145.58	15.64	110.00	192.00	28.58	2.04	24.00	32.00	19.12	2.95	12.00	27.00
*R. tanezumi*	34	162.91	10.46	128.00	180.00	173.09	16.06	144.00	205.00	31.25	1.72	28.00	35.00	21.72	2.24	18.00	26.00
*R. andamanensis*	44	173.02	19.54	126.00	212.00	186.89	24.45	115.00	231.00	31.65	1.87	27.00	36.00	23.15	2.29	19.00	32.00
*R. norvegicus*	69	182.62	24.50	120.00	274.00	152.67	20.80	82.00	222.00	34.42	3.59	26.00	41.00	17.72	2.23	11.00	21.00
*R. exulans*	4	115.50	14.27	101.00	135.00	138.00	8.17	126.00	144.00	23.70	1.39	22.00	26.00	16.75	0.50	16.00	17.00
*R. rattus*	5	170.80	16.99	150.00	194.00	190.60	11.80	180.00	210.00	33.00	2.24	30.00	36.00	22.60	1.82	20.00	25.00

Abbreviations are explained in the Materials and Methods section. All measurements are in mm.

One-way ANOVA demonstrated significant differences (*P*<0.05) between the seven species in all external and cranial characters tested. Results showed significant differences as follows: SGL, NBL, ZB, SBL, UTRL, UMRL, ML, BL, HBL, HFL, and EL between *R. nitidus* and *R. losea*; NBL, UTRL, and HFL between *R. nitidus* and *R. tanezumi*; HBL and HFL between *R. nitidus* and *R. andamanensis*; BL, HBL, and EL between *R. nitidus* and *R. norvegicus*; SGL, NBL, ZB, SBL, UTRL, UMRL, ML, BL, HFL, and EL between *R. nitidus* and *R. exulans*; SGL, ZB, SBL, UMRL, HBL, HFL, and EL between *R. losea* and *R. tanezumi*; SGL, NBL, ZB, SBL, UTRL, UMRL, ML, BL, HBL, HFL, and EL between *R. losea* and *R. andamanensis*; SGL, NBL, ZB, SBL, UTRL, UMRL, ML, BL, and HFL between *R. losea* and *R. norvegicus*; ML, BL, and HFL between *R. losea* and *R. exulans*; in HBL, HFL, and EL between *R. losea* and *R. rattus*; UTRL between *R. tanezumi* and *R. andamanensis*; UTRL, ML, BL, HBL, HFL, and EL between *R. tanezumi* and *R. norvegicus*; SGL, NBL, ZB, SBL, UTRL, UMRL, ML, BL, HBL, HFL, and EL between *R. tanezumi* and *R. exulans*; HBL, HFL, and EL between *R. andamanensis* and *R. norvegicus*; SGL, NBL, ZB, SBL, UTRL, UMRL, ML, BL, HBL, HFL, and EL between *R. andamanensis* and *R. exulans*; UMRL between *R. andamanensis* and *R. rattus*; SGL, NBL, ZB, SBL, UTRL, UMRL, ABL, ML, and HFL between *R. norvegicus* and *R. exulans*; HBL and EL between *R. norvegicus* and *R. rattus*; and SGL, SBL, BL, HBL, HFL, and EL between *R. exulans* and *R. rattus*. Thus, the 12 morphological characters separated the seven species of *Rattus* and validated their occurrence in China.

When all individuals of the two subspecies of *R. nitidus* were subjected to an independent sample *t*-test for each variable, significant differences appeared in UTRL, UMRL, ML, and TL between *R. nitidus nitidus* and *R. nitidus* from Xizang.

### Molecular analysis

We obtained cyt *b* sequences for 78 specimens of *Apodemus* and 106 specimens of *Rattus*. *De novo* sequences were deposited in GenBank under accession Nos. MG748165–MG748348 (Supplementary Table S3).

Cyt *b* K2P interspecies distances for *Apodemus* ranged from 5.4% to 20.7% (Supplementary Table S4). The smallest distance occurred between *A. uralensis* and *A. pallipes*, and largest between *A. sylvaticus* and *A. latronum*. The distances for *Rattus* ranged from 2.1% to 16.5% (Supplementary Table S5). The smallest distance occurred between *R. baluensis* and *R. tiomanicus*, and the largest between *R. leucopus* and *R. argentiventer*. The K2P distance of *R. nitidus* from Xizang and *R. nitidus nitidus* was 0.019.

#### Matrilineal genealogy (haplotype phylogeny) of *Apodemus*

**Figure 4:**
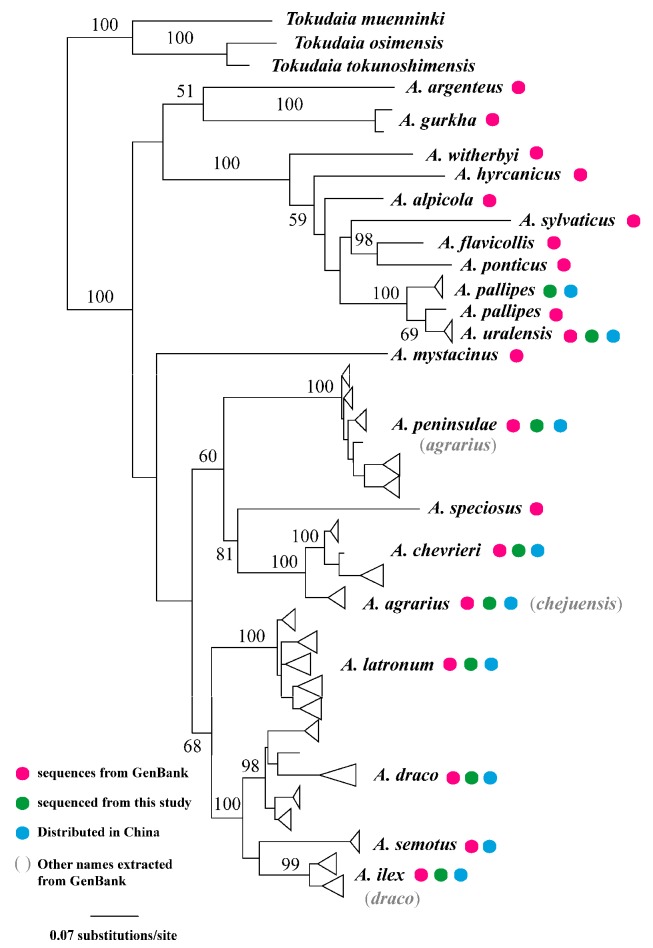
ML matrilineal genealogy of *Apodemus* derived from cyt *b* (Numbers above branches refer to bootstrap probabilities)

Matrilineal genealogy using the mitogenome and cyt *b* data for *Apodemus* (*n*=569) did not fully resolve the higher relationships (Figure 4), as in previous studies (see Discussion). Representative animals from China fell into nine clades that corresponded to nine species. Notably, *A. uralensis* from Xinjiang, China, fell into a clade (BS=100) comprised of *A. pallipes* from Xizang, China, and a sequence from GenBank (origin unknown), thus rendering *A. pallipes* paraphyletic (BS=69). A sole mitogenome representing “*A. chejuensis*” from Jeju Island was embedded in a clade containing *A. agrarius*. *Apodemus draco*, *A. ilex*, and *A. semotus* fell together in a well-supported clade (BS=100), but the relationships among the three species were not resolved (BS<50). *Apodemus* chevrieri, *A. draco*, *A. ilex*, *A. latronum*, and *A. peninsulae* also comprised subclades.

#### Matrilineal genealogy of *Rattus*

**Figure 5:**
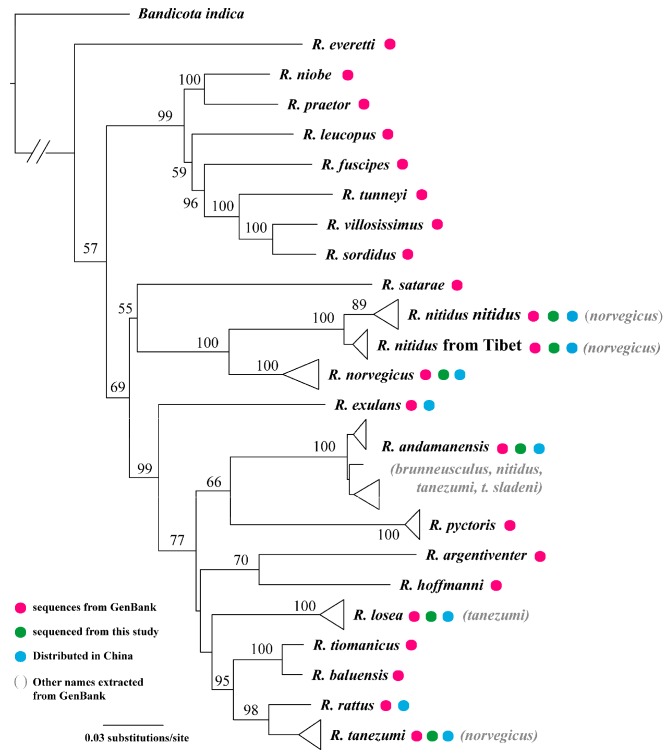
ML matrilineal genealogy of *Rattus* derived from cyt *b* (Numbers above branches refer to bootstrap probabilities)

The interspecific relationships of *Rattus* using the mitogenome and cyt *b* sequences (*n*=396) were well-resolved (BS=95–100) or moderately resolved (BS=55–77) (Figure 5). Sequences representing animals from China fell into seven lineages that corresponded with *R. nitidus*, *R. norvegicus*, *R. exulans*, *R. andamanensis*, *R. losea*, *R. rattus*, and *R. tanezumi*. The clade of *R. nitidus* had two subclades, one from southern Xizang and the other from southeastern China (Figure 5). The tree depicted GenBank sequences deposited under different names within a shallow clade, most commonly with *R. andamanensis*. However, some specimens were also associated with *R. losea*, *R. nitidus*, *R. tanezumi* as well as *R. nitidus* from southern Xizang.

## DISCUSSION

### Genealogy and taxonomy

Species of *Apodemus* are among the most destructive of all animal pests, yet little attention has been paid to their evolutionary relationships. Our trees were consistent with those from the robust study of Steppan & Schenk (2017), indicating the repeatability of both. However, the created molecular phylogenetic tree of *Rattus* was inconsistent with that of Aplin et al. (2011), which may be due to the different ways in which the trees were constructed (ML phylogeny here, but BI and NJ methods in Aplin et al. (2011)), different number of species, or different sequences of the cyt *b* gene (only two individuals of *R. pyctoris* (GenBank accession No. JN675511 and JN675512) from Aplin et al. (2011)). The unresolved relationships within *Apodemus* were not surprising and are likely due to early radiation in the evolution of this genus, as indicated by the saturation of the mitochondrial gene (Serizawa et al., 2000). Similar problems likely also occur in *Rattus* due to hybridization and introgression. Previously, *Rattus* was recovered as a paraphyletic genus (Steppan & Schenk, 2017). Fully resolved phylogenies require many slowly evolving and unlinked genes, which is not within the scope of the current study.

Despite uncertainty in phylogenetic relationships, questions regarding taxonomy in both genera remain. The differences between *A. pallipes* and *A. uralensis* have been discussed previously in depth (Musser & Carleton, 2005). Our carefully identified specimens of *A. pallipes* were from southern Xizang (Pulan County). The average cyt *b* genetic distance between *A. uralensis* and *A. pallipes* was 5.4%, which was the smallest interspecific genetic distance in *Apodemus*. All our specimens of *A. pallipes* matched the original description and holotype (Musser & Carleton, 2005). Thus, *A. pallipes* undoubtedly occurs in China. The sequences of *A. pallipes* in GenBank were from Afghanistan and Pakistan, near the type locality of *A. pallipes* in Pamir Alta. However, as we had no access to these specimens, it was not possible to determine if they matched the morphological description of *A. pallipes*.

Johnson & Jones (1955) described *A. chejuensis*. Koh (1991) also recognized the species based on its large body size and mtDNA genotype. Corbet (1978) assigned it as a synonym of *A. agrarius* ningpoensis, whereas Musser & Carleton (2005) treated it as a synonym of *A. agrarius*. Our phylogeny embedded *A. chejuensis* in *A. agrarius*, and thus our results agree with the assignment of Musser & Carleton (2005).

The taxonomic statues of *A. draco* remains uncertain. *Apodemus ilex* and *A. semotus* are close relatives to each other (Figure 4). Kaneko (2011) suggested that *A. semotus* did not differ significantly from *A. draco*. However, this endemic species of Taiwan was characterized by a dark gray pelage rather than the reddish-brown color of all other Asian species of *Apodemus*. Further, our ANOVA results demonstrated significant differences in TL and HFL between *A. ilex* and *A. draco*. Thus, we recognize all three as full species to better reflect their long evolutionary histories and distinct distribution patterns. Nevertheless, future comprehensive morphological diagnosis is desirable.

Hodgson described *R. pyctoris* in 1845 from Nepal (Hodgson, 1845). This name was later replaced by *R. rattoides* or *R. turkestanicus*. Musser & Carleton (1993) resurrected the oldest name and it has been reported to occur in China (Allen, 1940; Corbet, 1978; Ellerman & Morrison-Scott, 1951; Musser & Carleton, 1993, 2005; Wang, 2003). Feng et al. (1986) identified a series of specimens of *R. pyctoris* from Xizang and claimed that *R. pyctoris* closely resembled *R. rattus* but with a pale underbelly, relatively long nasal bone, and cusp t3 on M^1^. Our series of specimens from Xizang coincide with the characteristics of *R. pyctoris* described by Feng et al. (1986). However, phylogenetic analysis associated the species with *R. nitidus*. The original description and comments of Musser & Carleton (2005) on *R. pyctoris* point to its diagnostic characters as a very small cusp t3 on M^1^, a wide and short rostrum (narrow and slender in *R. nitidus*), and chunky wide molars (thinner and gracile in *R. nitidus*). Except for the morphology of M^1^, the Xizang specimens differed from *R. pyctoris*. Furthermore, many characters of the Xizang specimens also differed from *R. nitidus*, including the cusp t3 being present, gray-white underbelly, and larger measurements. The molecular phylogeny also placed the Xizang specimens and *R. nitidus* in different clades. Accordingly, we assign the Xizang specimens to a new, undescribed subspecies of *R. nitidus*.

Peale (1848) described *R. exulans* from Society Island. Nevertheless, its existence in Taiwan, China has been recognized for a long time (Motokawa et al., 2001). The Guangdong Insects Institute collected specimens of *R. exulans* from Yongxing Island in 1975. *Rattus exulans* is the smallest Asian species in its genus. The specimens from Yongxing Island conformed to the characteristics of *R. exulans*. Thus, we confirm that *R. exulans* occurs in China in Yongxing Island and Taiwan.

The earliest Chinese specimen of *R. rattus* (black type) was collected by A. B. Howell from Kuliang, Fukien in 1929 (Allen, 1940). In 1955 and 1956, the Fujian Epidemic Prevention Station collected specimens from Fujian, which were confirmed by Shou (1962) as being *R. rattus*. Our examination of these specimens and one specimen from Guangdong Province resulted in the same conclusion. Thus, we confirm that *R. rattus* occurs in Fujian and Guangdong.

### Morphometrics- and molecular-based species identifications

Regardless of skull and external measurements being similar between species, many interspecies measurements differed significantly. Species of *Apodemus* were easier to identify than *Rattus*. Furthermore, the different species of *Apodemus* exhibited stronger geographic distribution. For example, although measurements could not discriminate between *A. draco* and *A. ilex* (current study) or *A. semotus* (Kaneko, 2011), all three were found to be allopatric: *A. ilex* occurs in Hengduan Mountains, south of the Yangtze River and west of the Jinsha River; *A. semotus* occurs in Taiwan only; and *A. draco* occurs in the middle and lower reaches of the Yangtze River and in eastern China. *Apodemus chevrieri*, *A. draco*, and *A. latronum* co-occur in western Sichuan, but they were separated by the third upper molar and certain measurements (Figure 2A, B). Only one sequence of *A. peninsulae* in GenBank was likely misidentified (assuming no other error). Thus, the confusion between *A. draco* and *A. ilex* appears to be due to out-of-date taxonomy rather than misidentification.

Identification of *Rattus* species using either morphometrics or molecular data requires caution. Unlike for the species of *Apodemus*, most species of *Rattus* are invasive in China and have likely experienced strong selection resulting in morphological modification to adapt to local habitats. Notwithstanding, it was possible to identify some species based on morphology alone, such as, *R. andamanensis*, which has a unique white belly, *R. norvegicus*, which has very short ears, and *R. nitidus* and *R. norvegicus*, which do not have the cusp t3 on M^1^, with the former also having distinctly larger ears. The Chinese population of *R. rattus* is black all over its body, whereas *R. exulans* only occurs in islands of the South China Sea, including Taiwan, and has a very small head and body length. However, *R. losea* and *R. tanezumi* occur sympatrically in southern China. They are easily confused due to similar appearances and overlapping measurements. Most species showed significant overlap in the PCA plots (Figure 3A, B). Perhaps due to challenges in identification, GenBank contains many misidentifications. For example, sequences under the name of *R. norvegicus* occur in almost all clades (Figure 5).

Our new sampling and survey of sequences supported the occurrence of nine species of *Apodemus* and seven species of *Rattus* in China. However, it is necessary to be cautious with morphometric and molecular analyses for species identification due to considerable intraspecific variation and considerable errors in GenBank.

### Alpha diversity of *Apodemus* and *Rattus*

We determined that *A. agrarius, A. chevrieri, A. draco, A. ilex, A. latronum, A. pallipes, A. peninsulae, A. semotus*, and *A. uralensis* occur in China. In addition, considerable intraspecific diversity occurs in several species. Future comprehensive and integrative analyses can determine if further splitting is necessary and/or desirable.

We determined that *R. andamanensis, R. exulans, R. losea*, *R. nitidus*, *R. norvegicus*, *R. rattus*, and *R. tanezumi* occur in China. Future research into the occurrence of *R. pyctoris* in China is not necessary. A new subspecies of *R. nitidus* is described as follows:

### Subspecies description

#### *Rattus nitidus thibetanus* subsp. nov

**Holotype**: Adult female, collected by Liao Rui on 15 January 2011. The specimen was prepared as a skin with cleaned skull and deposited in the Sichuan Academy of Forestry (MT11197).

**Type locality**: Motuo County, Xizang, China, N29.24344° and E95.169920°, 783 m a.s.l..

**Measurements of holotype**: Weight: 179.6 g; HBL: 205 mm; TL: 200 mm; HFL: 40 mm; EL: 23 mm; SGL: 45.49 mm; SBL: 43.00 mm; ZB: 19.98 mm; MB: 17.06 mm; ABL: 8.08 mm; LMxT: 7.38 mm; NBL: 19.00 mm.

**Paratypes**: 5 specimens, with skins and skulls: XCY01001, ♀, 28.5048, 97.01045; XZ16259, ♀; 27.47033, 88.91450; XZ16258, ♂; 27.47033, 88.91450; MT020, ♂; MT035, ♂, 29.25491, 95.21331.

**Additional specimens**: 15 specimens (9 juveniles, 6 adults with skulls broken). Adults: XZ16260, ♀; XZ16253, ♂; XZ11280, ♀; XZ11262, ♂; XZ11177, ♂; and XZ11173, ♀. Juveniles: XZ11207, ♀; MT11174, ♀; XZ11176, ♀; XZ11207, ♀; XZ11208, ♀; XZ11232, ♀; XZ11175, ♀; XZ11196, ♂; and XZ11028, ♀.

**Geographic distribution**: The new subspecies is recorded from Yadong, Motuo, Nielamu, and Jilong counties, southern Xizang, China.

**Etymology**: The name is derived from the type locality, southern Xizang (Tibet), China.

**Diagnosis**: Cusp t3 present on M^1^ in first transverse loop, but very small; head and body relatively large; tail length usually larger than head plus body length; belly gray-white; transition between darker dorsal and lighter ventral pelage abrupt; dorsum of feet white, not glossy.

**Description**: Summer pelage from neck to hip uniform brown-black. Ventral hairs with gray-black base and gray-white tip, transition between darker dorsal and lighter ventral pelage relatively abrupt. Dorsal and ventral tail uniform brown-black; hairs on dorsal and venter of feet white, not glossy.

**Figure 6:**
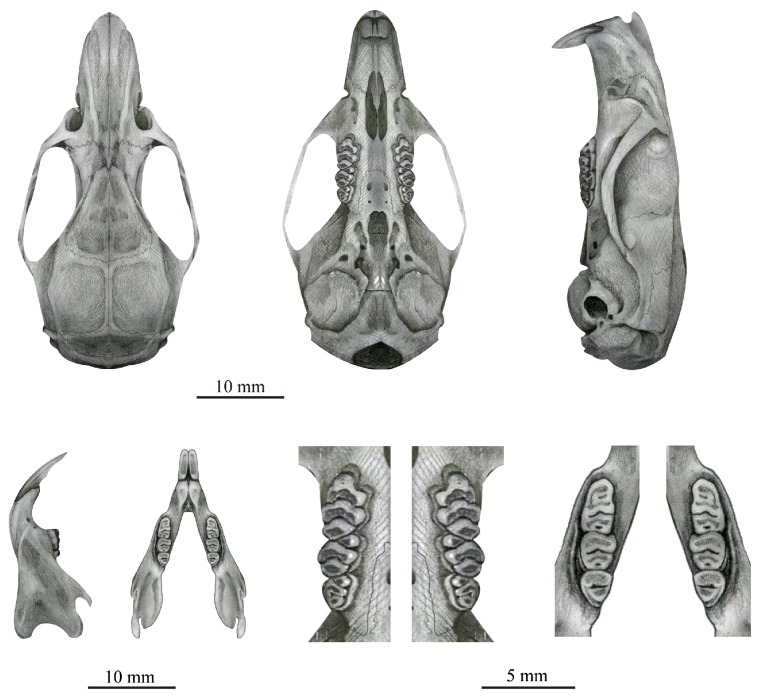
Skull of new subspecies of *Rattus nitidus*

Skull sturdy (Figure 6), in dorsal profile straight and brain case flattened; highest point of skull in middle of parietal bone. Nasal broad anteriorly narrowing posteriorly. Posterior margin of nasals irregular and protruding in front of maxilla. Posterior and anterior of frontal broad, middle narrower. Interparietal broad, anterior part triangle-shaped and posterior margin arc-shaped (Figure 6). Interorbital and temporal ridges present. Zygomatic arches medium in size, front part slightly broader. Auditory bullae moderately sized. Incisory foramen broad. Mandibles medium-sized (Figure 6).

Upper incisors medium in size vertically downward and orange. Molars rooted; 1st upper molar with three transverse dental loops, first dental loop with 3 cusps, t3 present but small; 2nd upper molar with three transverse dental loops, first dental loop only on lingual cusp; 3rd upper molar with three transverse dental loops, first dental loop only on lingual cusp, third loop only single semicircle and second loop rectangular; mandibular condyle and coronoid process large, but lower molar same as in other species of *Rattus*.

**Habitat**: Specimens were collected from an abandoned farmland, along the footpath of a rice field where highland barley was grown, forest edge, shrubland, surrounding a house, and salvage station.

**Comparison with other subspecies**: Compared with *R. n. nitidus*, t3 of the first dental loop present in *Rattus nitidus thibetanus* **subsp. nov** (vs. t3 absent or just vestigial; belly gray-white, and transition between darker dorsal and lighter ventral pelage relatively abrupt in *Rattus nitidus thibetanus* **subsp. nov** (vs. belly gray-white or yellow-gray, and transition vague in *R. n. nitidus*); dorsum of feet white, not glossy in *Rattus nitidus thibetanus* **subsp. nov** (vs. dorsum of feet white and shiny pearl in *R. n. nitidus*). The independent sample *t*-test demonstrated significant differences in UTRL, UMRL, ML, and TL between *R. n. nitidus* and *R. n. thibetanus*. The K2P distance for *R. n. thibetanus* and *R. n. nitidus* was 0.019, smaller than the smallest interspecies distance known in *Rattus*.

## Supplementary Material

http://www.zoores.ac.cn/fileup/2095-8137/SUPPL/20180720192342.zip
